# Oral Health Related Behaviors in Relation to DMFT Indexes of Teenagers in an Urban Area of North-West Poland—Dental Caries Is Still a Common Problem

**DOI:** 10.3390/ijerph18052333

**Published:** 2021-02-27

**Authors:** Marta Milona, Joanna Janiszewska-Olszowska, Monika Szmidt, Karolina Kłoda, Tomasz Olszowski

**Affiliations:** 1 Department of Hygiene and Epidemiology, Pomeranian Medical University in Szczecin, 70-111 Szczecin, Poland; tomasz.olszowski@pum.edu.pl; 2 Department of Interdisciplinary Dentistry, Pomeranian Medical University in Szczecin, 70-111 Szczecin, Poland; jjo@pum.edu.pl; 3 Department of Conservative Dentistry with Endodontics, Pomeranian Medical University in Szczecin, 70-111 Szczecin, Poland; monika@inet.pl; 4 Medfit Karolina Kłoda, 70-240 Szczecin, Poland; wikarla@gazeta.pl

**Keywords:** adolescents, caries, oral hygiene, teenagers, urban area

## Abstract

Caries has a negative influence on health and is still a public health problem among children and adolescents in Poland. The aim of this study was to analyze the association of dietary habits, oral hygiene behaviors and the frequency of usage of dental services with the dental caries index in teenagers in North-West Poland. The study enrolled 264 children (147M/117F) aged 15. Participants filled out a questionnaire regarding age, sex, frequency of visits to the dentist, dietary habits and oral hygiene behaviors, and the Decayed Missing Filled Teeth Index (DMFT) was calculated. Caries was found in 88.6% of subjects. The lower or no caries experience group (DMFT ≤ 5) comprised of 180 subjects, while higher caries experience (DMFT > 5) was found in 84 teenagers and was significantly inversely associated with tooth brushing after the last meal (OR = 0.45; 95% CI:0.21–0.97; *p* = 0.04) and the daily use of dental floss (OR = 0.12; 95% CI:0.01–0.92; *p* = 0.04). There is an emerging need for the implementation of effective caries prevention and recovery programs in Poland. Health promotion focusing on oral hygiene behaviors should be disseminated more widely because lower caries experience was demonstrated in teenagers declaring healthy oral habits. Another important need is the development of multi-sectorial actions aiming at the improvement of dietary habits.

## 1. Introduction

Caries is the most prevalent disease amongst children and adolescents, which has a negative influence on health [1]. In the second half of the 20th century, it was considered to be an infectious disease. Nowadays, it is regarded to have a complex nature with an important role of the biofilm and behavioral as well as with socio-economic factors (or confounders) which influence the likelihood for lesion development [2]. Thus, this multifactorial nature requires the implementation of effective preventive measures at social, school, family, individual and population levels [3].

Dental caries, gingival diseases and periodontal diseases are chronic and occur commonly in children, but are preventable [4,5]. These conditions are more prevalent within marginalized populations and disadvantaged communities [6]. At an individual level, risk factors include dietary patterns, oral health behaviors and practices, together with the use of dental care. The composition of meals and the frequency of food intake are considered to be the strongest factors associated with the cariogenic effect of the diet. The frequent consumption of products containing sugars, especially between meals, contributes to the increase in caries prevalence, particularly in people with poor oral hygiene [7,8,9]. Modern preventive programs are directed at children and adolescents and focus on the formation of correct eating habits, including the limitation of sugar consumption together with the promotion of anti-plaque cleaning activities. The most important are regular tooth brushing with fluoride toothpaste and cleaning the proximal surfaces of the teeth [10,11].

For many years, there has been no complex dental prophylaxis program in Poland, enabling children and young people to obtain at least, discussed below, an average oral health level for the European Union (EU). The Polish Government statistic materials show that the frequency of dental caries in 12-year-old children is 85%, and their Decayed Missing Filled Teeth (DMFT) index is 3.8, while in 15-year-olds it is 94% and their DMFT is 5.65 [12]. These are some of the worst results in the EU.

The aim of the present study was to analyze the association of dietary habits, oral hygiene behaviors and the frequency of usage of dental services with the dental caries index in teenagers in North-West Poland.

## 2. Materials and Methods

The study comprised of 264 children (147 boys and 117 girls) aged 15, attending public lower secondary schools in the system of obligatory general education, in the city of Szczecin, northwestern Poland. Schools (*n* = 10) included in the study were located in different districts (North, South, East, West, Downtown) and were randomly drawn from the list of public gymnasiums (*n* = 37). Such a list was placed on the website of the Department of Education of the Szczecin Municipal Office under the link “List of public schools and educational units in Szczecin”. The randomization was performed with use of a random numbers table. In the second stage of the procedure, one class, out of the whole year, was selected by the same method. The protocol of the study was approved by the Bioethics Committee of the Pomeranian Medical University (decision reference No. KB-0012/81/14).

Each participant and their guardian gave written consent for participation in this study. Other details concerning the study subjects’ recruitment and their clinical dental examination have been described in our previous paper [13]. The Decayed Missing Filled Teeth Index (DMFT) was calculated in every child as the sum of decayed (D), missing due to caries (M) and filled teeth (F). This was performed by visual-tactile examination [14]. Adolescents were examined by the same dentist and the test result was marked directly on the individual study card. The detection of open cavities was based on visual inspection of the dental surfaces with the aid of a dental lamp and dental mirror. A dental probe was used to remove plaque that could be covering a lesion, and then the blunt side of the probe was used to assess the surface roughness and to check for signs of enamel breakdown. The white spot lesion was considered as a healthy tooth. Children with moderate or high caries experience with a DMFT > 5 were assigned to the “higher caries experience” group, while those with low or very low caries experience (DMFT ≤ 5) were assigned to the “lower caries experience” group [15]. The Significant Caries Index (SiC Index) was calculated as follows: individuals were sorted according to their DMFT values. One third of the population with the highest caries scores were selected, according to Bratthl [16]. The mean DMFT for this subgroup was calculated. In addition, all the teenagers filled out a questionnaire containing questions about age, sex, frequency of visits to the dentist, dietary habits and oral hygiene behaviors. Dietary habits analyzed included the frequency of consumption of sweets, sweet drinks and snacks in separate ranges: at all, less frequently than once a week, once a week, 2–4 times a week, once a day, more often than once a day. Health behaviors related to oral hygiene have been evaluated based on answers to questions about the time and frequency of brushing teeth, the use of dental floss, and the use of interdental brushes, irrigators, toothpicks and mouthwashes. The entire questionnaire is listed in Table 1.

The tap water in Szczecin, which is drinkable, is not artificially fluoridated: the mean fluoride concentration ranges from 0.11 to 0.25 mg/L (data obtained from the Municipal Sanitary Epidemiological Inspectorate).

The results were statistically analyzed using Statistica 11 software (TIBCO Software Inc., Palo Alto, CA, USA). The comparisons of the median values of the index, according to gender, were made using the Mann–Whitney test. The evaluation of the statistical significance of differences between mean frequencies was generated using the chi-square test with Yates correction factor or the Fisher’s exact test. The logistic regression method was performed to identify the predictors of dental caries experience using a free statistics program [17]. The backward elimination method was used to select variables for the logistic regression model. The level of significance has been established at *p* = 0.05.

## 3. Results

Prevalence and caries experience (DMFT and SiC) in the studied 15-year-olds are presented in Table 2.

In the present study, caries was found in 88.3% of subjects. In males, the mean DMFT was 4.1 and in females it was 4.2, with no significant difference (*p* = 0.78). Moderate and high caries (DMFT > 5) experience was found in 84 (31.8%) teenagers (31.2% males and 32.4% females), while low or very low caries experience (DMFT ≤ 5) was found in 180 (68.2%) subjects. The SiC index was 7.5, not differing significantly between the sexes. Oral health behaviors in relation to caries experience are presented in Table 3.

Most teenagers (82%) brushed their teeth at least twice a day. Females, as compared to males, had significantly higher odds of performing oral hygiene habits, such as toothbrushing twice a day (OR = 11.68; 95% CI: 4.05–33.66; *p* = 0.000001) and brushing their teeth after their last meal before bedtime (OR = 9.74; 95% CI: 2.89–32.82; *p* = 0.00008) (see Table 3). There was no statistical significant difference between the sexes (*p* = 0.13) regarding toothbrush replacement. The frequency of toothbrush replacement (at least every 3 months) had no influence on DMFT (*p* = 0.16). Similarly, subjects toothbrushing at least twice a day did not have a significantly lower DMFT than those brushing their teeth less frequently (*p* = 0.12). In order to maintain oral hygiene, the teenagers examined used mouthwash (54.9%) and dental floss (45%) the most often (see Table 3). Dental floss was used statistically more often by the girls (OR = 2.36; 95% CI: 1.44–3.89; *p* = 0.0009), and the boys were more likely to reach for toothpicks (OR = 0.38; 95% CI: 0.18–0.82; *p* = 0.02) (Table 4). The logistic regression model revealed two significant predictors of “higher” caries experience: tooth brushing after the last meal and the daily use of dental floss (Table 5). Among the examined teenagers, “higher” caries experience (DMFT > 5) was significantly inversely associated with toothbrushing after the last meal (OR = 0.45; 95% CI: 0.21–0.97; *p* = 0.04) and the daily use of dental floss (OR = 0.12; 95% CI: 0.01–0.92; *p* = 0.04). Over 40% of adolescents ate sweets at least once a day, and 15.5% drank sweet sodas at least once a day. Girls (73.5%), more often than boys (54.4%), limited the drinking of sugary soft drinks to at most one day a week (*p* = 0.002). Subjects eating sweets (*p* = 0.19) or drinking soft drinks (*p* = 0.08) once a week or less frequently were not characterized by a significantly lower DMFT (see Table 3). Only 57.9% of the surveyed youth reported regularly for dental check-ups, and 18.6% declared that they visit the dental office only in the case of pain or when they noticed a cavity in a tooth. There were no significant differences in reporting for dental visits referring to gender or caries experience. Gender differences in selected oral health behaviors and dietary habits in a group of 15-year-old teenagers in Szczecin are presented in Table 4.

## 4. Discussion

This study focused on the association of dietary habits, oral hygiene behaviors and the frequency of usage of dental services with the dental caries index in teenagers inhabiting North-West Poland. Alarming observations from our study show that caries is still a common problem in this part of Central Europe. We have confirmed that the oral hygiene behaviors of the studied adolescents are of tremendous importance for their oral health. Tooth brushing after the last meal and the daily use of dental floss were the two significant inverse predictors of “higher” caries experience. Moreover, poor dietary habits (over 40% ate sweets at least once a day) and inadequate usage of dental service (18.6% visit the dental office only in the case of pain or noticing a cavity) are additionally contributing to the amount of 88.3% of teenagers with caries in our study.

The age of adolescence is important for the formation of positive oral hygiene habits that would be replicated in adult life. It is also a time when children start presenting oppositional behaviors towards parents and teachers, which may result in difficulties in effective health education [18]. Therefore, educational programs promoting desirable oral health behaviors are most effective if they are directed to children (and their parents) and people involved in their daily lives from the earliest school years. These programs must take into account the change of attitudes, behaviors and knowledge through a multi-level approach with the simultaneous wide use of social support [19,20].

The oral health of children and adolescents in Poland, despite the improvement at the turn of the 20th and 21st centuries, has remained at a low level for a decade [21]. The dental care system in Poland guarantees free prevention to children, but dentists rarely use preventive interventions (professional tooth cleaning, individual use of fluoride, dietary advice, determining the level of oral hygiene, individual oral hygiene instructions) in practice [22]. While there is a lack of continuous, common, integrated and multi-faceted programs of oral health promotion that allow introducing and maintaining changes to teenagers’ attitudes and behaviors, it is impossible to achieve oral health levels similar to their peers in EU countries [23]. In our study, 15-year-old children from North-West Poland were characterized by a lower DMFT compared to the result of nationwide data (4.1 vs. 5.8 (2015) or 6.1 (2011)), but the prevalence of caries was the same (88.3% vs. 91.8%) [24,25]. In comparison to such countries as Sweden, Germany or Great Britain, we have almost twice as many (88.6%) 15-year-olds with obvious decay experience, and the DMFT indicator is almost three times higher (Sweden 1.2, Germany and Great Britain 1.4) [26,27]. In Great Britain, only 9% of respondents reported high caries experience (DMFT > 5), while in our study, 31% of teenagers reported high caries experience. In Swiss 15-year-olds, the SiC index was similar (4.39) to the level of our DMFT indicator (4.1) [28,29]. Lower rates have also been achieved by teenagers from Portugal (DMFT 2.5 and 32% caries free), Greece (DMFT 3.19 and 29% caries free) and Romania (DMFT 3.6 and 21.5% caries free), as well as SOS Children’s Village residents in Croatia (DMFT 1.61) [30,31,32,33]. A prevalence of caries similar to Poland in this age group was found in the youth of Bulgaria (91.5%), Latvia (92.9%) and the Czech Republic (86.4%), with a DMFT of 5.0 in both the Czech Republic and Bulgaria, and 5.5 in Latvia [34].

Positive associations between free sugars intake and dental caries were found in all age groups (including <5 years to >65 years); in developing, transitional and industrialized countries. In general, the evidence suggests a positive relationship between the amount of free sugars consumption and dental caries, both in children and adults. According to a WHO report, the recommendation for free sugars intake was changed from the previous 10% (WHO, 2003) to 5% of daily energy intake to reduce obesity and dental caries [8]. Free sugars include monosaccharides and disaccharides added to foods and beverages by the manufacturer, cook or consumer, and sugars that are naturally present in honey, syrups, fruit juices and fruit juice concentrates. As dental caries is the result of lifelong exposure to a dietary risk factor (free sugars), even a small reduction in the risk of dental caries in childhood is of significance to future dental health. Therefore, to minimize the lifelong risk of dental caries, free sugars intake should be as low as possible [35,36]. However, the mean sugar consumption in EU countries is high at 39.0 kg/person/year, and in Poland, it is high at 44.1 kg/year (2012 data) [37]. Many authors show a relationship between the frequency of consumption of sugary drinks/confection and enamel erosion [38,39,40].

There are only a few studies on the oral health behaviors of Polish teenagers (age 12–18 years), and unfortunately, commonly available knowledge is not reflected in their health habits. Only less than a quarter of the surveyed teenagers (23.5%) in the present study limited their consumption of sweets to at most one day a week. Subjects with DMFT < 5 limited their consumption of sweets more often (26.1%) than those with high carious activity (17.9%). We observed a similar trend when limiting sweet carbonated beverages: 62.9% of the respondents drank them once a week or less. A higher percentage of girls than boys (73.5% vs. 54%) and a higher percentage of children with lower caries experience compared than those with DMFT > 5 (66.7% vs. 54.8%) reported drinking sweet carbonated beverages once a week or less. On the other hand, the percentage of young people who declared daily consumption of sweets was alarmingly high (42.4%). Even against the background of the Polish authors’ research, it was a very high result. In the nationwide survey it amounted to 25.3%, and among teenagers from the Mazovian region, 22.8%. Sugary drinks in our study were drunk by 15.5% teenagers daily compared to 20% in the nationwide survey and 17.7% in the Mazovian region [24,41]. The advantage of our study in comparison to earlier research is assessing not only oral health behaviors and dietary habits, but also performing clinical examination and associating the obtained findings. Moreover, data from North-West Poland were lacking.

School shops, prevalent in our country, may have had an influence in the strengthening of these negative habits. Eighty-eight percent of pupils had access to school shops in their schools, and their assortment was often limited to items considered highly cariogenic: confectionery (iced donuts, sweet cakes), sweets and chips, and sweetened beverages. Since September 2015, the Polish Government has introduced strict restrictions on the distribution of products containing sugar and sweeteners as defined in the Regulation of the European Parliament and Council No. 1333/2008, and on food additives in educational institutions, completely excluding confectionery and semi-confectionery products, sweetened breakfast cereals, and processed fruit and vegetables with the addition of sugar or sweeteners. Unfortunately, only one year later these restrictions were withdrawn [42,43]. This is the opposite direction to the recommendation regarding providing children in schools and pre-schools with access to meals and beverages, taking into account the latest health recommendations, and promoting a reduction in processed food [44]. It is therefore difficult to popularize the model of eating sweets one day a week by children and young people, which is widespread in many countries, especially Scandinavian countries. Due to this model, only 17% of twelve-year-olds in Finland and 14% of eighteen-year-olds in Norway eat sweets every day [45,46].

Despite the obvious evidence that the consumption of larger amounts of total sugars, sugar-containing foods/beverages as well as the greater frequency of consumption of sugar-containing foods/beverages, but not the frequency of consumption of total sugars, is associated with a greater risk of dental caries, the importance of sugars as a cause of caries is not prominent in preventive strategies. This is despite overwhelming evidence of its unique role in causing a worldwide caries epidemic. The long-standing failure to identify the need for drastic national reductions in sugar intakes reflects scientific confusion, partly induced by pressure from major industrial sugar interests [47,48]. Unfortunately, Poland is listed among the countries with a high percentage of the population affected by dental caries and also by being overweight or obese.

Plaque is considered the most important risk factor for caries. Effective oral hygiene is a key factor in maintaining good oral health, and tooth brushing with fluoride toothpaste is thought the most important [49,50]. The addition of dental floss and an interdental brush to brushing effectively removes the plaque from the interdental spaces. This will allow a reduction in oral hygiene indexes, and it significantly reduces the occurrence of periodontal diseases [11,51]. According to our research, the percentage of 15-year-olds in Szczecin who brushed their teeth at least twice a day was surprisingly very high and amounted to 82%, but only 7% used dental floss daily. However, in our study, the daily use of dental floss (OR 0.12; *p* = 0.04) and tooth brushing after the last meal before bedtime (OR 0.45; *p* = 0.04) proved to be significant factors lowering the risk of higher caries experience (DMFT > 5). Similarly, Julihn et al. found that irregular toothbrushing in the evening was strongly associated with a high caries prevalence in 19-year-olds [52]. Additionally, recent evidence supports a recommendation that brushing with a fluoride toothpaste should take place just prior to going to bed; fluoride concentrations in saliva 12 h after brushing directly before bedtime were comparable with those found 1–4 h after brushing during the day [53].

According to the latest data, brushing teeth just as often as twice a day was declared by young people from Sweden (77–86% girls and 71% boys) and Scotland (83% girls and 65% boys) [54,55]. Definitely lower rates of youths who brushed their teeth twice or more times a day were recorded in the Czech Republic (79.5% girls and 61.9% boys), in France (70.9%), in Portugal (65.1%) and in Australia (57%) [56,57,58,59]. In accordance with the results of other authors, we observed a significant difference between the genders referring to the frequency of toothbrushing [59,60,61]. Girls were brushing their teeth at least twice a day (96.6%, *p* = 0.0001) and before bedtime (97.4%, *p* = 0.0001) more often than boys. They used dental floss more often (56.4%, *p* = 0.001) as well. Additionally, in another study of 15-year-old youths from central Poland, the percentage of people brushing their teeth at least twice a day was lower (73.6%) compared to the result of our study. However, the percentage of teenagers who used floss was similar (49.5% vs. 45%) [41], but studies by Polish authors in other regions showed even lower proportions of junior high school students using dental floss: 37% [62] and 12.9% [63]. The positive results we obtained in terms of the declared toothbrushing twice a day can be a sign of the superior knowledge of recommended/desirable health behaviors amongst our teenagers, but they do not necessarily reflect the actual attitudes.

Many authors believe that the attitude towards the use of dental services is dependent on economic conditions, the level of education and cultural determinants [64,65]. Early prevention and diagnosis of caries is a result of regular visits to a dentist. There was a significant relationship between the date of the first visit of a child and the costs of future treatment (i.e., an increase in costs with the time of the first visit being delayed) [66]. A visit to the dentist not only has a therapeutic aspect, but educational intervention conducted by health professionals, in the context of their practice, helps to promote oral health in the population [67,68,69,70]. Spanish children regularly attending dental visits had lower caries indexes (1.76) than children attending them irregularly (2.5) [71]. Meanwhile, the fear of a visit to the dentist is common in society and results from the fact that dentistry is a surgical specialty, and carious disease seems impossible to avoid [72,73]. Our results confirm the thesis about the reluctance to attend regular meetings with the dentist. It was declared by only 57% of teenagers we examined, and almost every fifth (18.6%) visited the dentist only out of pain or a visible cavity. This is also confirmed by the Dudek study [41], in which over half of junior high school students (58.0%) visited the dentist every 6 months, and 17.4% of children visited a dentist only in the case of acute pain. The mean examination interval of youths below 17-years-old in Finland was 1 year, while in New Zealand, 79.9% of adolescents reported having visited a dentist during the last year [74,75].

## 5. Conclusions

Since the dental status of 15-year-old teenagers in Poland is characterized by high values of the DMFT index, it is necessary to implement effective prevention and recovery programs. Health promotion focusing on oral hygiene behaviors should be disseminated more widely because lower caries experience was demonstrated in teenagers declaring tooth brushing after their last meal before bedtime and the daily use of dental floss. Another important area is the development of multi-sectorial actions aiming at the improvement of dietary habits, due to the fact that almost half of the examined adolescents declared daily consumption of sweets.

## Figures and Tables

**Table 1:** The questionnaire form.

1	How often do you brush your teeth? less than once a day once a day twice a day after each meal
2	In the morning I brush my teeth: before breakfast after breakfast I do not brush my teeth at all I just rinse my mouth
3	After the last evening meal before bedtime I do not perform any oral hygiene procedures I just rinse my mouth I brush my teeth thoroughly
4	I use for oral hygiene additionally (apart from toothbrush and toothpaste) dental floss interdental toothbrush irrigator mouthwash toothpicks none
5	I use dental floss never less than once a week 1–4 times a week once a day more than once a day
6	I replace my toothbrush once a year every 6 months every 3 months every month
7	I eat sweets never less than once a week once a week 2–4 times a week once a day more than once a day
8	I eat snacks, breadsticks, crackers never less than once a week once a week 2–4 times a week once a day more than once a day
9	I drink soft drinks never less than once a week once a week 2–4 times a week once a day more than once a day
10	I visit the dental office only when I feel toothache only when I notice a cavity in my tooth every 6 months

**Table 2:** Caries experience (DMFT, SiC) among 15-year-old teenagers in Szczecin.

	Total N 264 (100%)	Males N 147 (100 %)	Females N 117 (100%)	*p* Value *
Subjects with caries experience (DMFT > 0)	233 (88.3%)	131 (89.1%)	102 (87.2%)	
Mean DMFT	4.1	4.1	4.2	0.78
Number of subjects with moderate and high caries experience (DMFT > 5)	84 (31.8%)	46 (31.2%)	38 (32.4%)	
SiC	7.5	7.4	7.6	0.8

* Mann–Whitney test, DMFT—Decayed Missing Filled Teeth Index, SiC—Significant Caries Index.

**Table 3:** Oral health behaviors and dietary habits in relation to caries experience of 15-year-old teenagers in Szczecin.

	Total N = 264 (100%)	DMFT ≤ 5N = 180 (100%)	DMFT > 5N = 84 (100%)	*p* Value *
**Tooth brushing**				
twice daily or more often	217 (82%)	153 (85%)	64 (76.2%)	0.12
after breakfast	172 (65.1%)	121 (67.2%)	51 (60.7%)	0.37
after last meal before bedtime	231 (87.5%)	163 (91%)	68 (81%)	0.05
toothbrush replacement at least every 3 months	204 (77.2%)	144 (80%)	60 (71.4%)	0.16
**The use of auxiliary oral hygiene aids**				
dental floss	118 (45%)	86 (47.8%)	32 (38%)	0.18
dental floss every day	19 (7%)	18 (10%)	1 (1.2%)	0.01
mouthwash	145 (54.9%)	99 (55%)	46 (54%)	0.92
toothpicks	39 (15.9%)	27 (15%)	12 (14.3%)	0.99
**Dietary habits**				
sweets once a week or less	62 (23.5%)	47 (26.1%)	15 (17.9%)	0.19
sweets once daily or more	112 (42.4%)	72 (40%)	40 (47.6%)	0.3
snacks once a week or less	212 (80.3%)	145 (80.6%)	67 (79.8%)	0.99
snacks once daily or more	16 (6.1%)	12 (6.7%)	4 (4.8%)	0.74
soft drinks once a week or less	166 (62.9%)	120 (66.7%)	46 (54.8%)	0.08
soft drinks once daily or more	41 (15.5%)	29 (16.1%)	12 (14.3%)	0.84
**Visiting the dentist**				
just in case of pain or when I notice a cavity in a tooth	49 (18.6%)	34 (18.9%)	15 (17.8%)	0.98
control visit once every six months	153 (57.9%)	109 (60.5%)	44 (52.4%)	0.26

* chi square test with Yates correction factor, DMFT—Decayed Missing Filled Teeth Index.

**Table 4:** Gender differences (Females vs. Males) in selected oral health and dietary habit behaviors of 15-year-old teenagers in Szczecin.

Risk Factor	OR (95%CI)	*p* Value *
Tooth brushing twice or more per day	11.68 (4.05–33.66)	0.000001
Tooth brushing after last meal before bedtime	9.74 (2.89–32.82)	0.00008
Using dental floss	2.36 (1.44–3.89)	0.0009
Using toothpick	0.38 (0.18–0.82)	0.02
Soft drinks once a week or less	2.32 (1.34–4.07)	0.002

* Fisher exact test.

**Table 5:** Logistic regression model describing the predictors of “higher” caries experience (DMFT > 5) among the examined 15-year-old teenagers in Szczecin.

Variable	OR (95%CI)	*p* Value
Tooth brushing after last meal before bedtime	0.45 (0.21–0.97)	0.04
Using dental floss every day	0.12 (0.01–0.92)	0.04

Overall Model Fit Chi square = 12.79 *p* = 0.001.
